# Individual-level brain phenotypes in first-episode mania: normative modelling of brain morphometry and brainAGE

**DOI:** 10.1192/bjo.2025.28

**Published:** 2025-05-09

**Authors:** Kevin Yu, Ruiyang Ge, Yuetong Yu, Shalaila Haas, Nicole Sanford, Lakshmi N. Yatham, Sophia Frangou, Trisha Chakrabarty

**Affiliations:** Department of Psychiatry, Djavad Mowafaghian Centre for Brain Health, University of British Columbia, Vancouver, BC, Canada; Department of Psychiatry, Icahn School of Medicine at Mount Sinai, New York, NY, USA

**Keywords:** Bipolar disorder, brainAGE, first-episode mania, structural MRI

## Abstract

**Background:**

Brain morphological alterations in bipolar disorder are well documented, particularly in chronic cases. This study focuses on first-episode mania (FEM) to quantify neuroanatomical changes at early stages of the disorder.

**Aims:**

To assess deviations from normative brain morphometry and age-related brain changes in patients with FEM.

**Method:**

Pretrained models, based on large, independent healthy samples, were applied to structural brain images from FEM patients (*n* = 83) and healthy individuals (*n* = 61). Normative deviation *z*-scores were computed for regional brain morphometry, along with global and voxel-level brain–age-gap estimates (G-brainAGE and L-brainAGE, respectively). The proportions of infranormal (*z* < −1.96) and supranormal (*z* > 1.96) deviations were measured for both groups. Ridge regression and support vector machine models were used to evaluate whether *z*-scores predicted symptom severity, IQ or diagnosis. Case-control differences in L-brainAGE and correlations between G-brainAGE and clinical features were analysed.

**Results:**

Both FEM and healthy individuals showed similar proportions of infra- and supranormal deviations in regional measures. Morphometric data, whether observed or normative, did not significantly predict clinical outcomes or diagnosis. Mean G-brainAGE in FEM was −1.04 (s.d. 3.26) years and negatively correlated with age of onset, while L-brainAGE did not differ significantly between groups.

**Conclusions:**

Regional morphometry and local brain-ageing metrics in FEM patients aligned with normative ranges, suggesting minimal abnormalities in early bipolar disorder. However, subtle delays in global brain ageing may reflect variation based on the age of onset, highlighting a potential area for further exploration.

Bipolar disorder is a severe mental illness characterised by depressive and manic symptoms,^1^ and is a significant contributor to the global burden of disease.^2^ A rich neuroimaging literature has identified morphological alterations in bipolar disorder compared with healthy individuals, primarily in brain regions involved in the integration of emotional and cognitive processing (e.g. anterior cingulate, prefrontal cortex, hippocampus, amygdala, thalamus and striatum).^3–6^ The current study moves away from traditional group comparisons to investigate brain structure in first-episode mania (FEM) using normative models of regional morphometry and of brain ageing, which offer personalised assessments of the integrity of brain organisation.^7,8^ Normative modelling maps the variation in neuroimaging measures within a healthy population, enabling the consistent assessment of deviations from typical patterns.^9^ Normative modelling of brain morphometry has been successfully used to dissect individual-level abnormalities in regional neuroanatomy in multiple psychiatric disorders, but not yet in first-episode bipolar disorder.^7,10–12^ Brain morphometry data have also been used to establish a normative reference pattern for typical age-related brain changes.^9^ Large gaps between the biological and chronological age of an individual’s brain (brain–age-gap estimate, brainAGE) indicate atypical development or ageing.^13^ Several studies have reported accelerated ageing, as indexed by higher brainAGE, in patients with chronic bipolar disorder;^14–16^ by contrast, our group and others have reported negative brainAGE values in early-stage bipolar disorder, suggesting a delay in typical brain maturational trajectories at disease onset.^17,18^ These global indices of brainAGE (henceforth G-brainAGE), however, do not quantify regional patterns of brain ageing. Local voxel-based brainAGE (henceforth L-brainAGE) provides greater spatial granularity and has been used to identify different patterns of brain ageing in the early stages of psychosis, but has yet to be used in bipolar disorder.^19^ To address these gaps, the current study assessed the clinical utility and correlates of these individualised measures of regional morphometry and brain ageing in a unique sample of bipolar disorder patients recently recovered from their FEM. Our initial hypotheses were that: (a) patients with FEM would show greater divergence from normative patterns of morphometry in regions implicated in bipolar disorder by prior neuroimaging studies; (b) regional normative deviation scores would better predict clinical features and discriminate between patients and healthy comparators versus observed morphometric values; and (c) patients with FEM would show evidence of atypical brain ageing, as indexed by G-brainAGE and L-brainAGE.

## Method

### FEM sample

Patients meeting Diagnostic and Statistical Manual of Mental Disorders, Fourth Edition, Text Revision (DSM-IV-TR) criteria for bipolar I disorder (*N* = 83, mean age 22.3 years,^4,6^ age range 14–35 years) (Table 1) were enrolled in the Systematic Treatment Optimization Program for Early Mania (STOP-EM) from the University of British Columbia (UBC) Hospital and affiliated sites, and from community referrals. The complete protocol has been previously described.^17^ Inclusion criteria for patients with FEM were as follows: (a) age 14–35 years; (b) first manic/mixed episode in the 3 months prior to enrollment; (c) receiving mood-stabilising treatment and clinically stable at enrollment; (d) no history of brain injury or neurological disorder; and (e) sufficient English proficiency to follow study procedures. A comparison sample of demographically matched healthy individuals, without personal or family history of psychiatric disorders or neurological disorders/brain injury (*N* = 61, mean age 23.8 years (s.d. 5.05), age range 16–37 years), were recruited using similar procedures. All participants were of normal weight based on body mass index (BMI) of 19–24. FEM patients and healthy individuals did not differ in regard to age (*P* = 0.26), BMI (*P* = 0.87), sex distribution (*P* = 0.92) or general cognitive ability (*P* = 0.15). The study was approved by the UBC Clinical Research Ethics Board, and written informed consent was obtained from all participants prior to enrollment.

**Table 1:** Characteristics of the first-episode mania patient sample (*N* = 83)

Age in years, mean (s.d.)	22.18 (4.63)
Female sex, *N* (%)	44 (53.01)
Estimated IQ, mean (s.d.)	105.63 (7.75)
YMRS total score, mean (s.d.)	1.59 (3.29)
MADRS total score, mean (s.d.)	5.77 (6.96)
Age of onset (years), mean (s.d.)	16.87 (8.14)
Duration of illness (years), mean (s.d.)	2.80 (4.16)
Unmedicated patients, *N* (%)	5 (6.02)
Patients prescribed lithium monotherapy, *N* (%)	0 (0)
Patients prescribed adjunct lithium, *N* (%)	38 (45.78)
Patients prescribed valproate, *N* (%)	31 (37.35)
Patients prescribed antipsychotics, *N* (%)	60 (72.29)
Patients prescribed antidepressants, *N* (%)	7 (8.43)
Patients prescribed benzodiazepines, *N* (%)	10 (12.05)
Patients prescribed >1 psychotropic, *N* (%)	70 (84.34)

YMRS, Young Mania Rating Scale; MADRS, Montgomery–Åsberg Depression Rating Scale.

### Clinical assessment

Diagnostic status in all participants was established using the Mini International Neuropsychiatric Interview, administered by a board-certified psychiatrist.^20^ An estimate of general cognitive ability (IQ) in all participants was obtained using the North American Adult Reading Test (NAART).^21^ Information collected on patients at the time of scanning pertained to illness duration (defined as time in years from first mood episode – depressive, hypomanic or manic), age of onset (age at first mood episode), medication status and severity of manic and depressive symptoms as respectively assessed using the Montgomery–Åsberg Depression Rating Scale (MADRS)^22^ and Young Mania Rating Scale (YMRS).^23^

### Neuroimaging

Whole-brain T1-weighted data on all study participants were acquired using a Philips Achieva 3 Tesla scanner (Koninklijke Philips N.V., The Netherlands; Supplementary Material S1 available at https://doi.org/10.1192/bjo.2025.28). Images were processed using two different methods: (a) standard pipelines implemented in the FreeSurfer image analysis suite (http://surfer.nmr.mgh.harvard.edu/), yielding a total of 150 morphometric features (Table S1) comprising Desikan–Killiany atlas measures of cortical thickness (*n* = 68), cortical surface area (*n* = 68),^24^ regional subcortical volumes (*n* = 14) based on the Aseg atlas^25^ and an estimate of intracranial volume (ICV) (Supplementary Material S2). These measures were used in computing normative deviations and G-brainAGE in the study sample (Supplementary Material S3, S4 and S5); and (b) established procedures implemented with Statistical Parametric Mapping (SPM12; https://www.fil.ion.ucl.ac.uk/spm/software/spm12/) to derive grey matter and white matter maps as input features for L-brainAGE (Supplementary Material S2).

### Computation of normative deviation scores for regional brain morphometry

This analysis utilised CentileBrain, a validated framework for generating normative models of brain morphometry. CentileBrain was developed using data from an independent multisite sample of 37 407 healthy individuals (53.3% female, age range 3–90 years; Supplementary Material S4; see https://centilebrain.org/ for full model procedure and freely available code).^9,12^ Briefly, sex-specific models for each measure were generated using fractional polynomial regression, with Combat-GAM harmonisation applied to account for site effects.^26^ Global measures (intracranial volume, mean cortical thickness and mean surface area) were included in regional models; the CentileBrain model parameters were then applied to the current study sample. For each measure in each participant, a *z*-score representing the degree of normative deviation from the reference population was generated. *Z*-scores were calculated by subtracting the predicted from the observed value of that measure and dividing the difference by the root mean square error (RMSE) of the model.^27,28^ A positive or negative *z*-score indicates that the value of the morphometric measure is higher or lower, respectively, than the normative mean. Consistent with previous studies,^29^ regional *z*-scores were classified as infranormal when below *z* = −1.96 (below the fifth percentile) or supranormal when above *z* = 1.96 (above the 95th percentile). *Z*-values between −1.96 and +1.96 were classified as ‘within normal range’.

### Computation of G-BrainAGE

CentileBrain also provides sex-specific models to obtain estimates of G-brainAGE trained on Freesurfer outputs derived from a sample of 35 683 healthy individuals (53.59% female, age range 5–90 years).^29^ These models are also freely available through the CentileBrain Web platform (https://centilebrain.org/#/brainAGE2) (https://centilebrain.org/#/brainAge_global) (Supplementary Material S5). The parameters from the pretrained models were applied to the data from male and female participants of the current study to obtain G-brainAGE for each participant.^30^ Positive and negative G-brainAGE values indicate apparent acceleration or delay, respectively, in age-related brain structural changes.

### Computation of L-brainAGE

The modulated grey and white matter maps derived from SPM12 were entered into a convolutional neural network (U-Net; https://github.com/SebastianPopescu/U-NET-for-LocalBrainAge-prediction) pretrained in an independent sample of 4155 healthy individuals aged 18–90 years (Supplementary Material S6). The pretrained model parameters^19^ were applied to the study sample data to yield individualised voxel-based L-brainAGE maps.

### Statistical analyses

Statistical significance across all tests performed was set at a positive false discovery rate (PFDR) of 0.05 using the Benjamini–Hochberg false discovery rate correction for multiple comparisons. Group differences in sociodemographic features and G-brainAGE were identified using parametric or chi-square tests as indicated.

Analyses using the normative brain regional *z*-scores comprised:

- Calculating percentages of FEM patients and healthy individuals from the study sample with supra- or infranormal *z*-scores in any regional measure. The percentage of FEM individuals with supranormal values for region *X* was computed as: ((*N* of FEM individuals with supranormal values in *X*/*N* of FEM individuals) × 100). The same method was used to calculate the proportion of FEM with infranormal regional *z*-scores, and for all measures in healthy individuals.
- Assessing group differences in the proportion of individuals with supra- or infranormal *z*-scores using the two-proportions *z*-test (R version 4.1.2).
- Conducting a series of ridge regression analyses (*α* = 1) to predict IQ, YMRS and MADRS scores in patients with FEM, from either the observed brain morphometric measures or brain regional *z*-scores following previously published procedures.^23^ Model performance was assessed by tenfold cross-validation using RMSE, mean absolute error (MAE) and the correlation between observed and predicted outcomes averaged across folds. Statistical significance was estimated against a null distribution generated from models trained on 100 000 random permutations of the neuroimaging data. *P*-values were assigned as the proportion of permuted scoring metrics greater than or equal to the true estimates.
- Comparing the ability of the observed brain regional measures or regional *z*-scores to predict case-control status using a linear support vector machine (SVM) with tenfold cross-validation implemented in Python, version 3.8 (Python Software Foundation).

Independent-samples *t*-testing was used to compare G-brainAGE between patients with FEM taking or not taking lithium. Pearson’s correlation was used to assess associations between G-brainAGE and IQ, symptom severity, illness duration and age of onset in FEM patients. Case-control differences in L-brainAGE were identified using general linear models in SPM12, with age and sex as covariates, followed by sex-specific analyses.

## Results

### Infra- and supranormal deviations in brain morphometry

**Fig. 1:**
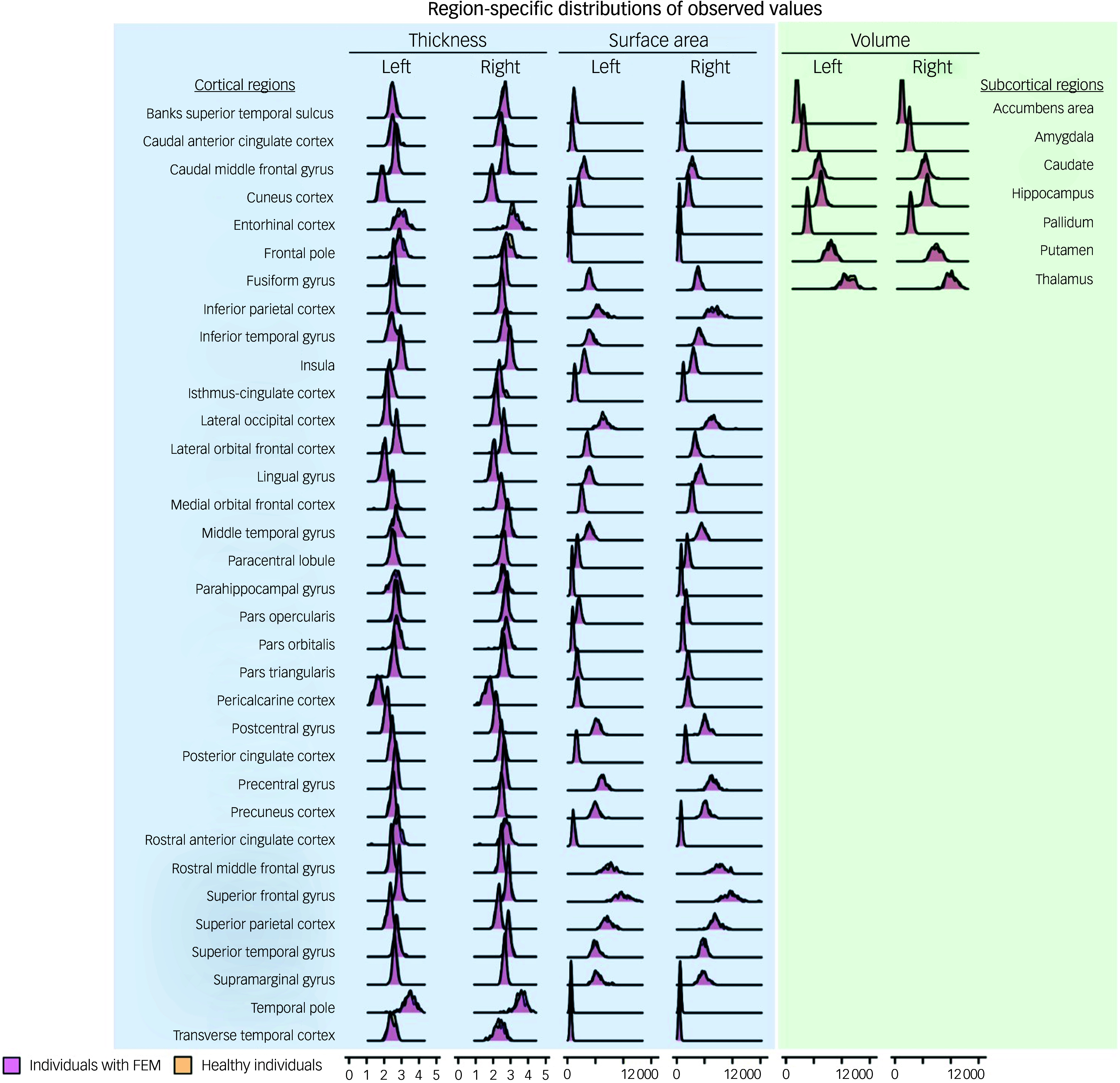
Distributions of observed regional morphometric measures in patients with first-episode mania (FEM) and healthy individuals.

**Fig. 2:**
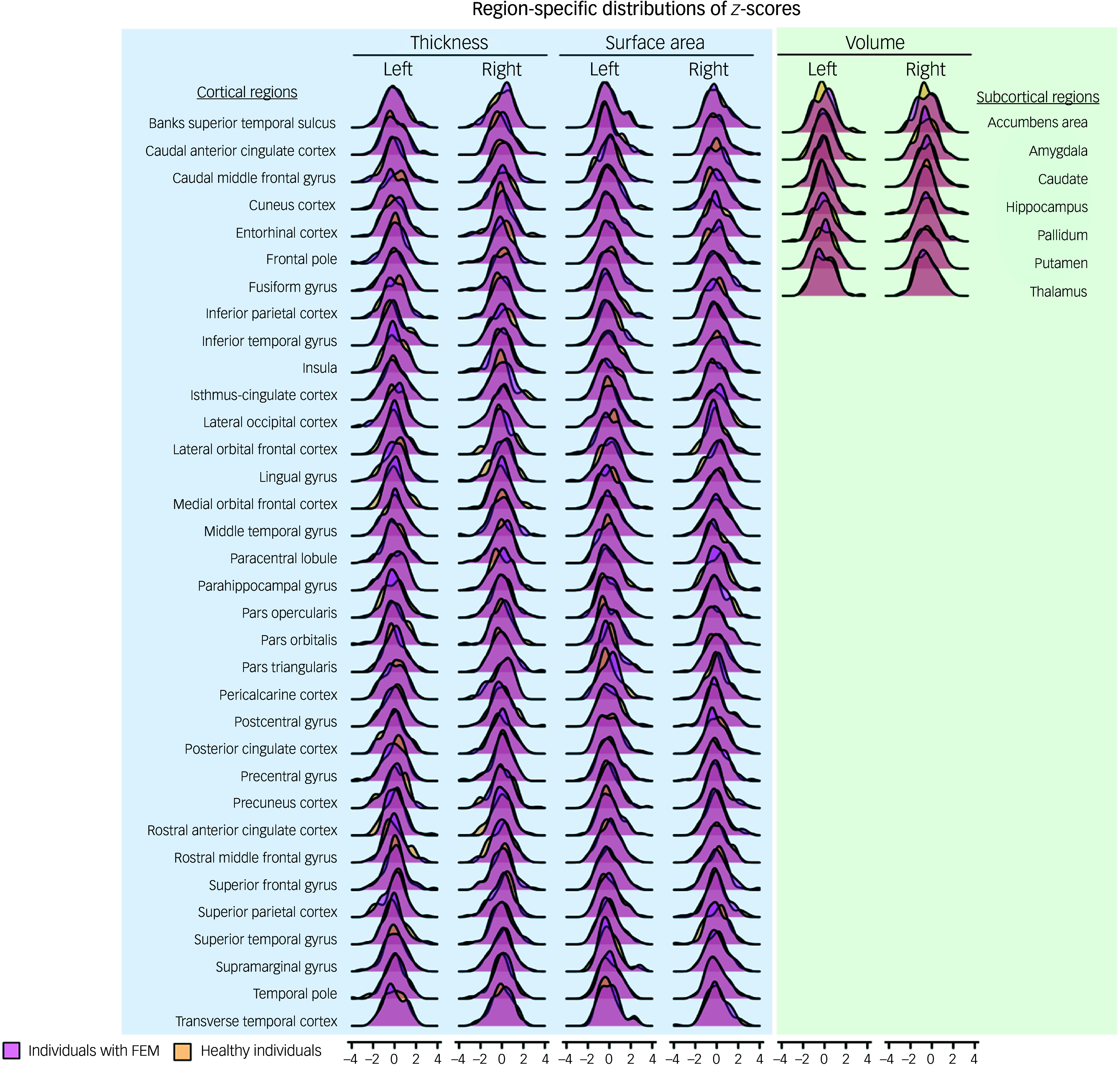
Distributions of regional *z*-scores in patients with first-episode mania (FEM) and healthy individuals.

**Fig. 3:**
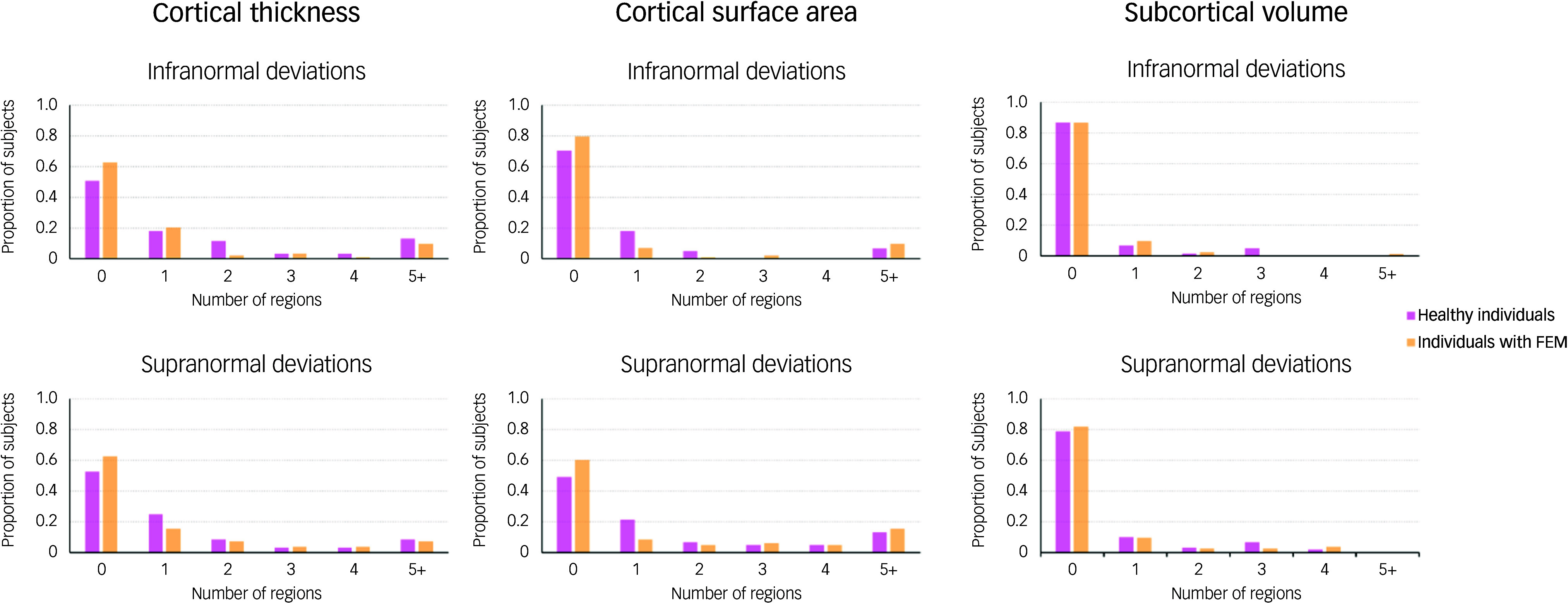
Distribution of the total number of regions with infra- or supranormal regional normative *z*-scores in patients with first-episode mania (FEM) and healthy individuals. Bar plots show the distribution of the total number of regions per individual, with infranormal (top) and supranormal (bottom) *z*-score in patients with FEM and in healthy individuals.

There was complete overlap in the distributions of *z*-scores and observed values of all regional morphometric measures of both patients with FEM and healthy individuals (Figs 1 and 2). The percentages of patients with FEM and healthy individuals with supra- or infranormal z-scores in each measure are shown in Supplementary Table S2. Infranormal regional cortical thickness *z*-scores were present in 0–6.02% of patients with FEM, and in 0–8.20% of healthy individuals; supranormal *z*-scores were present in 0–4.82% of patients with FEM and in 0-8.2% of healthy individuals. Infranormal regional surface area *z*-scores were present in 0–3.61% of patients with FEM and in 0–4.92% of healthy individuals, and supranormal *z*-scores were present in 0–7.23% of patients with FEM and in 0–8.2% of healthy individuals. Infranormal regional subcortical volume *z*-scores were seen in 1.20–3.61% of patients with FEM and in 0–4.92% of healthy individuals, and supranormal *z*-scores in 0–4.92% of FEM patients and in 0–4.82% of healthy individuals. There were no significant group differences in the percentage of individuals with infra- or supranormal regional values (PFDR > 0.05, Table S2). The proportion of patients with FEM that had one or more supra- or infranormal *z*-scores was comparable to that of healthy individuals (Fig. 3). Specifically, 41 individuals with FEM (49.40.%) had at least one infranormal *z*-score in any region, comparable to 39 healthy individuals (63.93%) with at least one infranormal *z*-score (*P* = 0.083). Supranormal *z*-scores in any region were observed in 52 individuals with FEM (62.65%), comparable to 47 healthy individuals (77.05%) with at least one supranormal *z*-score (*P* = 0.065). Additionally, there were no group differences in intracranial volume (*P* = 0.96).

### Predictive value of normative z-scores and observed regional morphometry values

**Fig. 4:**
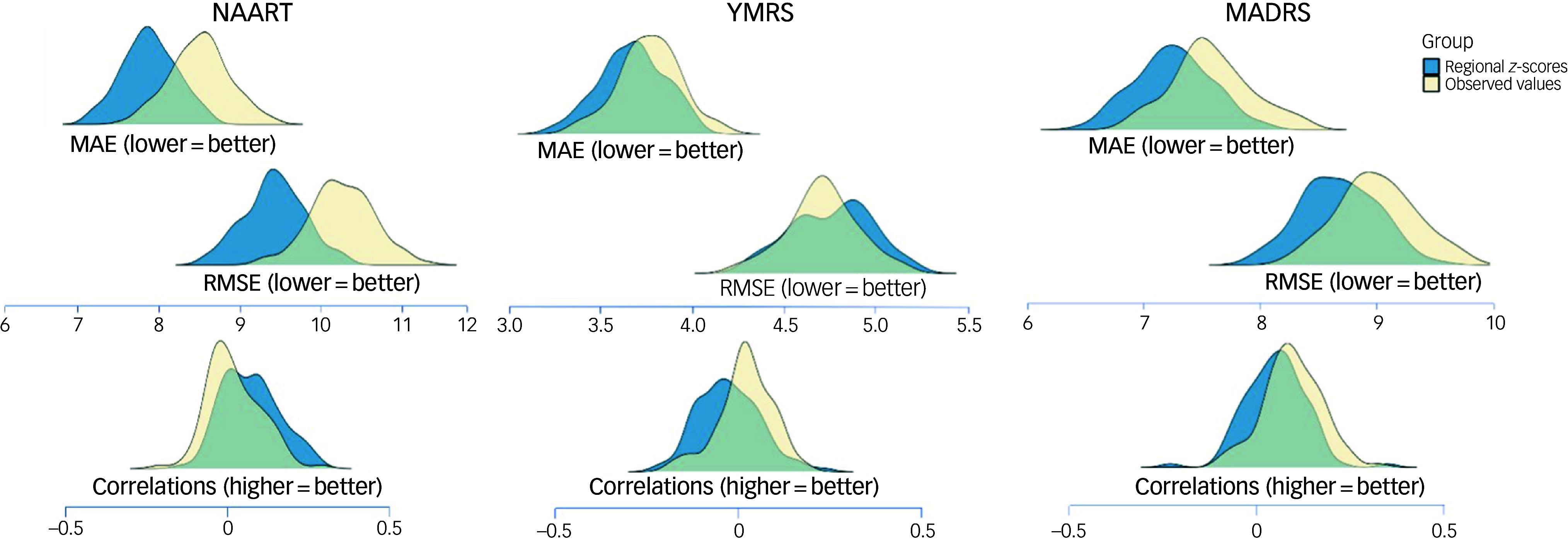
Predictive performance of regional *z*-scores and observed regional brain morphometric measures for psychopathology and general cognition in patients with first-episode mania. Separate ridge regression analyses were conducted for each outcome measure. Lower MAE and RMSE values (top) and higher correlation values (bottom) indicate better performance. MADRS, Montgomery–Åsberg Depression Rating Scale; MAE, mean absolute error; NAART, North American Adult Reading Test; RMSE, root mean square error; YMRS, Young Mania Rating Scale.

None of the ridge regression models for YMRS, MADRS or IQ were statistically significant, but the performance of the models using regional *z*-scores was numerically better in terms of lower MAE and RMSE and higher correlation values compared with models using observed morphometric measures (Fig. 4).

**Fig. 5:**
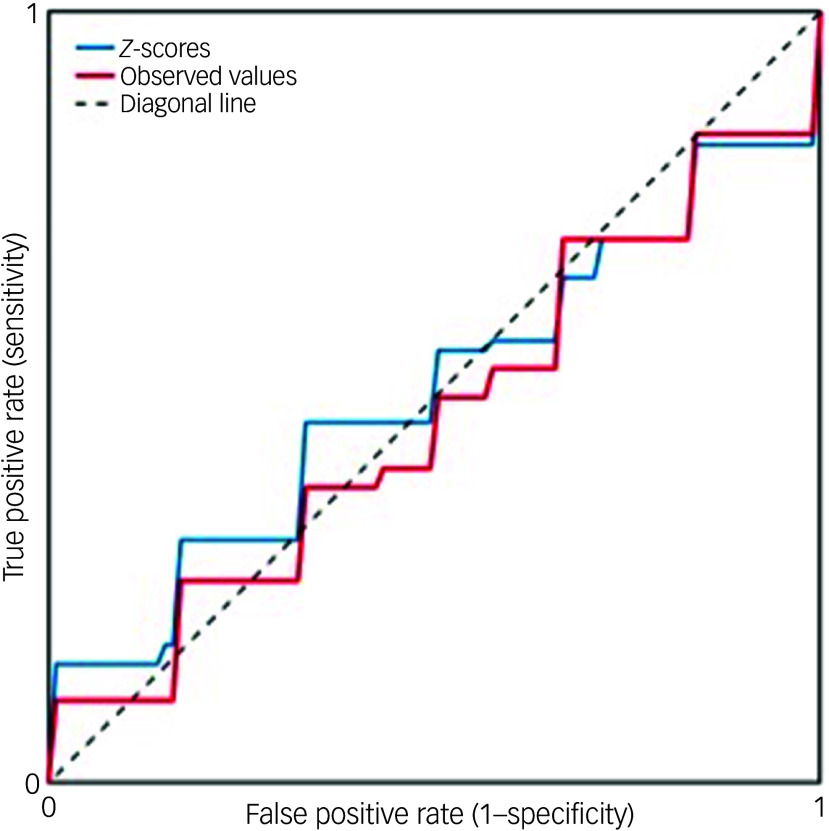
Receiver operator curves of models using either regional *z*-scores or observed regional brain morphometric measures for diagnostic classification. A linear support vector machine was applied to distinguish between patients and healthy individuals using either regional brain *z*-scores (indicated by the blue line, with area under the curve, AUC, 0.51) or observed morphometric measures (red line, with AUC 0.47).

SVMs using either the observed morphometric measures or normative *z*-scores were unable to distinguish between FEM and healthy individual cohorts (observed measures area under the curve (AUC) 0.47, *z*-score AUC 0.51; Fig. 5).

### Global and local brainAGE

The mean G-brainAGE of patients with FEM was −1.04 (s.d. 3.26) years, suggesting a slight delay in typical age-related structural changes in the brain across the entire patient sample. G-brainAGE in FEM individuals did not differ between sexes (*P* = 0.82) and was not associated with total YMRS score (*P* = 0.78, *r* = 0.032), MADRS (*P* = 0.64, *r* = −0.054) or IQ (*P* = 0.22, *r* = −0.138), or with duration of illness (*P* = 0.45, *r* = –0.089). G-brainAGE did not differ between FEM individuals on adjunctive lithium and those not on lithium (*P* = 0.798). There was a significant negative correlation between G-brainAGE and age of onset (*P* = 0.003, *r* = −0.350). To determine whether sex moderates this association, we conducted a linear regression with sex and age-of-onset as predictors of G-brainAGE; there was no significant main effect of sex (*P* = 0.75) or significant sex–age of onset interaction (*P* = 0.53).

Case-control differences in L-brainAGE were also not significant (Supplementary Material S7 and S8 and Fig. S1). Case-control comparisons with age and sex as covariates, as well as sex-specific analyses, were not significant at the designated PFDR < 0.05.

## Discussion

To our knowledge, this is the first study to jointly examine normative *z*-scores, G-brainAGE and L-brainAGE, the key available individualised measures of brain organisation, in FEM. In contrast to our initial hypotheses, the results suggest that both regional morphometry and local pace of brain ageing in patients with FEM are comparable to those of healthy individuals, indicating that regional alterations in the early course of bipolar disorder are minimal and have limited utility in predicting diagnosis or clinical features. However, the predictive models for categorical diagnosis or dimensional psychopathology and general cognition using normative *z*-scores performed numerically better than those using observed morphometric measures, highlighting the advantage of utilising individualised measures when brain alterations are very subtle. A global measure of brainAGE, representing an aggregate of regional brain-ageing patterns, suggested an overall slight deceleration from typical patterns of brain ageing in this FEM cohort.

The results indicate that variation in regional brain structure in patients with FEM is nested within typical variation. Extreme deviations from the normative mean were observed in each region in a small proportion of patients, comparable to those observed in healthy individuals. We have previously argued that, despite the power of advanced machine learning algorithms to differentiate cases from controls using structural neuroimaging data, the success of such efforts fundamentally depends on the degree of case-control similarity in neuroanatomical profiles.^31^ To this purpose, we have previously developed the person-based similarity index (PBSI) that can be applied to any clinical condition and, to date, has been used in bipolar disorder,^31^ schizophrenia^31,32^ and clinical high risk for psychosis.^33^ Examination of the PBSI in two independent samples of patients with bipolar disorder (total *N* = 122) has previously shown a very high degree of similarity in the neuroanatomical profiles of patients and healthy individuals, which was replicable and independent of medication.^31^ These findings are aligned with conventional case-control comparisons of brain morphometry, including voxel- or region-based analyses, which found minimal differences between patients with bipolar disorder and healthy individuals. For example, the largest case-control differences in subcortical volumes and cortical thickness have been found in the hippocampus^3^ and pars opercularis,^4^ but both are of small effect (Cohen’s *d* < 0.26). The current study adds to this literature and previous papers using normative models.^9,13^ Taken together, this evidence suggests that neuroanatomical alterations associated with bipolar disorder are generally small and are nested within normal variation, particularly at the early stages of the disorder as exemplified by the FEM sample used here. Consequent upon this, the predictive value of neuroanatomical measures for diagnosis or symptom dimensions is minimal.

G-brainAGE in patients with FEM indicated a slight delay from the expected rate of brain ageing, with no diagnostic differences in L-brainAGE. Previous individual studies on G-brainAGE in samples of patients with bipolar disorder with mixed and variable illness duration have been generally negative,^34,35^ although a meta-analysis (involving 938 patients)^14^ and a mega-analysis of G-brainAGE (involving 459 patients)^16^ reported subtle brain ageing acceleration of about +2 years. In this study, G-brainAGE showed a mean delay of −1.04 years, similar to previously reported G-brainAGE values in early-stage bipolar disorder.^17,18^ This includes an earlier analysis that calculated a G-brainAGE value of –1.00 in this FEM cohort using a model developed by the Enhancing NeuroImaging Genetics through Meta-analysis Consortium, supporting the model-independent reproducibility of the results.^17^ Slight deceleration in G-brainAGE has also been observed in people at familial risk for bipolar disorder, indicating that ‘younger’ G-brainAGE may be a vulnerability marker for bipolar disorder or a feature of the early stages of the disorder.^36^ It has been hypothesised that patients with bipolar disorder present with variable G-brainAGE depending on their exposure to the neuroprotective effect of lithium, as shown by Van Gestel and colleagues.^37^ However, in the present study sample, G-brainAGE did not differ between patients with FEM on lithium and those not on lithium. Additionally, atypical rates of brain ageing in bipolar disorder may be present in patients with medical comorbidities or pronounced cognitive dysfunction,^17^ although these would be a rather non-specific association because they are also observed in the general population.^38,39^ Different patterns of age-related morphological changes have also been observed in bipolar disorder patients with early (prior to age 25 years) versus late onset of mood symptoms.^40^ In the present FEM cohort, later age of onset was associated with lower G-brainAGE, supporting the hypothesis that time of disease onset in the developmental years may influence trajectories of brain ageing.

Several methodological issues are worth considering. A major strength of the study is that the individualised measures examined were tabulated using robust normative models generated from large and independent datasets of healthy individuals. Equally important is the inclusion of an exclusively FEM cohort. Limitations involve the cross-sectional nature of the data-set that does not inform on the possible evolution of deviations from normative reference trajectories. Further exploration of longitudinal associations among individualised brain measures and illness duration, episode recurrence, medication exposure, lifestyle factors, cognitive decline and physical health markers would be informative.

In conclusion, by demonstrating that individual-level brain phenotypes of patients with FEM fall within a normative range, this study serves as a starting point for future analyses of the longitudinal evolution of brain ageing and neuroanatomy in bipolar disorder. It also supports joint examination of individualised regional and global aggregate measures to understand the impact of disease-related factors on brain structure and ageing early in the course of bipolar disorder.

## Supporting information

Yu et al. supplementary material Yu et al. supplementary material

## Data Availability

The data that support the findings of this study are available from co-author L.N.Y. upon reasonable request.

## Supplementary material

The supplementary material is available online at https://doi.org/10.1192/bjo.2025.28
