# Supplementary material for: Individual-level brain phenotypes in first-episode mania: normative modelling of brain morphometry and brainAGE
Source: BJPsych Open. 2025 May 9;11(3):e95. doi: 10.1192/bjo.2025.28 (PMC12089812; doi:10.1192/bjo.2025.28)
Supplement: Yu et al. supplementary material [file S2056472425000286sup001.docx]

**Individual-level brain phenotypes in first episode mania: Normative modelling of brain morphometry and brainAGE**

**Supplementary Material**

**S1. Neuroimaging Acquisition and Quality Control**

**S2. Image Processing**

Table S1. FreeSurfer-Derived Brain Morphometric Measures

**S3. Proportion of Subjects with Infra- and Supranormal Regional Normative Z-Scores**

Table S2. Percentage of Individuals with Infra- or Supranormal Normative Regional Z-Scores Based on Group

**S4. Normative Modeling**

**S5. G-brainAGE Computation**

**S6. L-brainAGE Computation**

**S7. Group Differences in L-brainAGE**

Figure S1. Differences in L-brainAGE between healthy individuals and patients with first-episode mania**.**

**S8. Supplement References**

**S1. Neuroimaging Acquisition and Quality Control**

Whole-brain T1-weighted magnetic resonance images were acquired on a Philips Achieva 3.0 Tesla scanner (Koninklijke Philips N.V, The Netherlands) using a three-dimensional axial inversion recovery-weighted spoiled gradient recalled sequence and the following parameters: FOV = 20 cm (RL) × 25.6 cm (AP), ACQ matrix = 256 × 256, isotropic image voxels (1 × 1 × 1 mm3), autoshim, TR/TE = autoset shortest, T/R head coil, flip angle = 8 degrees, and 1 mm thick contiguous 180 slices of the whole brain.

Using visual inspection, each T1-weighted scan was assessed using four criteria: (1) image affected by movement, (2) temporal poles missing (even partly) in the reconstruction, (3) other parts of the cortex missing in the reconstruction, (4) non-brain tissue (e.g., dural/skull) still visible in the reconstructed pial surface. These criteria were applied separately for the left and right hemispheres. Each criterion was scored 0 if there were ‘no errors visible’ or 1 if there were ‘errors visible in at least three consecutive slices. Scans with scores of 1 or 2 were considered good quality; those with higher scores (>2) were considered of lower quality and were excluded.

**S2. Image Processing**

***S2.1 Reconstruction using FreeSurfer***: Cortical reconstruction and subcortical segmentation of the T1-weighted datasets were implemented in FreeSurfer image analysis suite (version 7.1.0; [http://surfer.nmr.mgh.harvard.edu/](http://surfer.nmr.mgh.harvard.edu)). Processing included removal of non-brain tissue using a hybrid watershed/surface deformation procedure, segmentation of the subcortical white matter and deep gray matter volumetric structures, intensity normalization, tessellation of the boundary of gray matter and white matter, automated topology correction and surface deformation following intensity gradients to optimally place the gray/white and gray/cerebrospinal fluid borders at the location where the greatest shift in intensity defines the transition to the other tissue class. The Desikan-Killiany atlas was used to parcellate the brain into 68 cortical regions [Desikan et al., 2006], while subcortical segmentation was performed using the probabilistic atlas in FreeSurfer [Fischl et al., 2004]. This procedure yielded measures of total intracranial volume (ICV), regional cortical thickness, surface area, and subcortical volumes (Table S1). Quality assessment of the FreeSurfer output used standardized ENIGMA procedures ([http://enigma.ini.usc.edu/protocols/imaging-protocols](http://enigma.ini.usc.edu/protocols/imaging-protocols/)).

| **Table S1. FreeSurfer Extracted Morphometric Measures** | | |
| --- | --- | --- |
| **Measure** | **Side** | **Regions** |
| **Cortical**  **Thickness** | **L** | left_bankssts, left_caudalanteriorcingulate, left_caudalmiddlefrontal, left_cuneus, left_entorhinal, left_fusiform, left_inferiorparietal, left_inferiortemporal, left_isthmuscingulate, left_lateraloccipital, left_lateralorbitofrontal, left_lingual, left_medialorbitofrontal, left_middletemporal, left_parahippocampal, left_paracentral, left_parsopercularis, left_parsorbitalis, left_parstriangularis, left_pericalcarine, left_postcentral, left_posteriorcingulate, left_precentral, left_precuneus, left_rostralanteriorcingulate, left_rostralmiddlefrontal, left_superiorfrontal, left_superiorparietal, left_superiortemporal, left_supramarginal, left_frontalpole, left_temporalpole, left_transversetemporal, left_insula |
|  | **R** | right_bankssts, right_caudalanteriorcingulate, right_caudalmiddlefrontal, right_cuneus, right_entorhinal, right_fusiform, right_inferiorparietal, right_inferiortemporal, right_isthmuscingulate, right_lateraloccipital, right_lateralorbitofrontal, right_lingual, right_medialorbitofrontal, right_middletemporal, right_parahippocampal, right_paracentral, right_parsopercularis, right_parsorbitalis, right_parstriangularis, right_pericalcarine, right_postcentral, right_posteriorcingulate, right_precentral, right_precuneus, right_rostralanteriorcingulate, right_rostralmiddlefrontal, right_superiorfrontal, right_superiorparietal, right_superiortemporal, right_supramarginal, right_frontalpole, right_temporalpole, right_transversetemporal, right_insula |
| **Surface Area** | **L** | left_bankssts, left_caudalanteriorcingulate, left_caudalmiddlefrontal, left_cuneus, left_entorhinal, left_fusiform, left_inferiorparietal, left_inferiortemporal, left_isthmuscingulate, left_lateraloccipital, left_lateralorbitofrontal, left_lingual, left_medialorbitofrontal, left_middletemporal, left_parahippocampal, left_paracentral, left_parsopercularis, left_parsorbitalis, left_parstriangularis, left_pericalcarine, left_postcentral, left_posteriorcingulate, left_precentral, left_precuneus, left_rostralanteriorcingulate, left_rostralmiddlefrontal, left_superiorfrontal, left_superiorparietal, left_superiortemporal, left_supramarginal, left_frontalpole, left_temporalpole, left_transversetemporal, left_insula |
|  | **R** | right_bankssts, right_caudalanteriorcingulate, right_caudalmiddlefrontal, right_cuneus, right_entorhinal, right_fusiform, right_inferiorparietal, right_inferiortemporal, right_isthmuscingulate, right_lateraloccipital, right_lateralorbitofrontal, right_lingual, right_medialorbitofrontal, right_middletemporal, right_parahippocampal, right_paracentral, right_parsopercularis, right_parsorbitalis, right_parstriangularis, right_pericalcarine, right_postcentral, right_posteriorcingulate, right_precentral, right_precuneus, right_rostralanteriorcingulate, right_rostralmiddlefrontal, right_superiorfrontal, right_superiorparietal, right_superiortemporal, right_supramarginal, right_frontalpole, right_temporalpole, right_transversetemporal, right_insula |
| **Subcortical**  **Volumes** | **L** | Left Thalamus, Left Caudate, Left Putamen, Left Pallidum, Left Hippocampus, Left Amygdala, Left Accumbens area |
|  | **R** | Right Thalamus, Right Caudate, Right Putamen, Right Pallidum, Right Hippocampus, Right Amygdala, Right Accumbens area |

***S2.2 Reconstruction using SPM12***: Preprocessing of the T1-weighted images was also carried out using standard pipelines implemented in the Statistical Parametric Mapping (SPM12) software package (https://www.fil.ion.ucl.ac.uk/spm/software/spm12/) to derive gray matter (GM) and white matter (WM) maps as input features for local brain age prediction. Images were normalized using affine followed by non-linear registration, corrected for bias field inhomogeneities, and segmented into GM, WM, and cerebrospinal fluid. Then, the Diffeomorphic Anatomic Registration Through Exponentiated Lie algebra algorithm (DARTEL) [Ashburner, 2007] was applied to normalize the segmented scans into a standard MNI space (MNI-152 space) which were then resampled to 1.5 mm3 with a 4mm smoothing kernel. These outputs were used for the computation of L-brainAGE.

**S3. Proportion of subjects with infra- and supranormal regional normative z-scores**

In order to identify the proportion of FEM with infra- and supranormal regional z-scores, for example, the percentage of FEM individuals with supranormal values for a region X was computed as: [(N of FEM individuals with supranormal values in X/N of FEM individuals)*100]. The same method was applied to identify the proportion of FEM with infranormal regional z-scores. The same was repeated for all measures in healthy individuals.

| **Table S2. Percentage of individuals with infra- or supranormal normative regional z-scores based on group** | | | | | |
| --- | --- | --- | --- | --- | --- |
|  |  | **Normative z-scores in HI** | | **Normative z-scores in FEM** | |
| **Region** | **Hemi** | **infranormal**  **z-scores (%)** | **supranormal**  **z-scores (%)** | **infranormal**  **z-scores (%)** | **supranormal**  **z-scores (%)** |
| **Subcortical Volume** | | | | | |
| nucleus accumbens | L | 1.64 | 3.28 | 2.41 | 2.41 |
|  | R | 3.28 | 4.92 | 1.20 | 2.41 |
| amygdala | L | 0.00 | 4.92 | 1.20 | 0.00 |
|  | R | 0.00 | 4.92 | 1.20 | 2.41 |
| caudate | L | 3.28 | 3.28 | 2.41 | 2.41 |
|  | R | 1.64 | 3.28 | 2.41 | 3.61 |
| hippocampus | L | 1.64 | 3.28 | 1.20 | 1.20 |
|  | R | 3.28 | 4.92 | 2.41 | 4.82 |
| pallidum | L | 0.00 | 3.28 | 1.20 | 4.82 |
|  | R | 4.92 | 1.64 | 2.41 | 3.61 |
| putamen | L | 1.64 | 0.00 | 1.20 | 2.41 |
|  | R | 1.64 | 1.64 | 2.41 | 1.20 |
| thalamus | L | 0.00 | 1.64 | 1.20 | 1.20 |
|  | R | 1.64 | 1.64 | 3.61 | 3.61 |
| **Cortical Thickness** | | | | | |
| banks superior temporal sulcus | L | 1.64 | 1.64 | 2.41 | 4.82 |
|  | R | 3.28 | 0.00 | 4.82 | 0.00 |
| caudal anterior cingulate | L | 1.64 | 3.28 | 1.20 | 1.20 |
|  | R | 0.00 | 3.28 | 0.00 | 3.61 |
| caudal middle frontal | L | 0.00 | 4.92 | 4.82 | 3.61 |
|  | R | 0.00 | 1.64 | 3.61 | 2.41 |
| cuneus | L | 1.64 | 0.00 | 0.00 | 2.41 |
|  | R | 4.92 | 0.00 | 2.41 | 2.41 |
| entorhinal cortex | L | 0.00 | 6.56 | 1.20 | 1.20 |
|  | R | 3.28 | 3.28 | 4.82 | 0.00 |
| fusiform gyrus | L | 3.28 | 1.64 | 2.41 | 1.20 |
|  | R | 3.28 | 1.64 | 2.41 | 1.20 |
| inferior parietal | L | 0.00 | 3.28 | 3.61 | 3.61 |
|  | R | 1.64 | 3.28 | 3.61 | 2.41 |
| inferior temporal | L | 0.00 | 1.64 | 0.00 | 4.82 |
|  | R | 1.64 | 0.00 | 2.41 | 2.41 |
| isthmus cingulate | L | 3.28 | 3.28 | 1.20 | 3.61 |
|  | R | 1.64 | 8.20 | 1.20 | 2.41 |
| lateral occipital | L | 3.28 | 1.64 | 4.82 | 1.20 |
|  | R | 3.28 | 1.64 | 3.61 | 1.20 |
| lateral orbitofrontal | L | 1.64 | 1.64 | 1.20 | 1.20 |
|  | R | 4.92 | 3.28 | 1.20 | 1.20 |
| lingual gyrus | L | 3.28 | 0.00 | 4.82 | 2.41 |
|  | R | 1.64 | 0.00 | 4.82 | 1.20 |
| medial orbitofrontal | L | 1.64 | 1.64 | 1.20 | 1.20 |
|  | R | 1.64 | 4.92 | 1.20 | 1.20 |
| middle temporal | L | 1.64 | 3.28 | 1.20 | 3.61 |
|  | R | 3.28 | 1.64 | 2.41 | 4.82 |
| parahippocampal | L | 4.92 | 0.00 | 3.61 | 0.00 |
|  | R | 1.64 | 4.92 | 2.41 | 0.00 |
| paracentral | L | 4.92 | 1.64 | 2.41 | 1.20 |
|  | R | 4.92 | 1.64 | 2.41 | 2.41 |
| pars opercularis | L | 0.00 | 3.28 | 2.41 | 3.61 |
|  | R | 1.64 | 3.28 | 2.41 | 4.82 |
| pars orbitalis | L | 3.28 | 0.00 | 2.41 | 1.20 |
|  | R | 1.64 | 3.28 | 2.41 | 0.00 |
| pars triangularis | L | 3.28 | 3.28 | 1.20 | 2.41 |
|  | R | 0.00 | 3.28 | 1.20 | 3.61 |
| pericalcarine | L | 0.00 | 3.28 | 1.20 | 1.20 |
|  | R | 3.28 | 0.00 | 1.20 | 0.00 |
| postcentral | L | 1.64 | 1.64 | 2.41 | 2.41 |
|  | R | 1.64 | 1.64 | 3.61 | 2.41 |
| posterior cingulate | L | 0.00 | 1.64 | 2.41 | 1.20 |
|  | R | 1.64 | 1.64 | 3.61 | 2.41 |
| precentral | L | 1.64 | 1.64 | 2.41 | 1.20 |
|  | R | 4.92 | 0.00 | 2.41 | 0.00 |
| precuneus | L | 3.28 | 0.00 | 2.41 | 4.82 |
|  | R | 8.20 | 0.00 | 3.61 | 2.41 |
| rostral anterior cingulate | L | 1.64 | 3.28 | 1.20 | 1.20 |
|  | R | 4.92 | 1.64 | 1.20 | 1.20 |
| rostral middle frontal | L | 1.64 | 1.64 | 3.61 | 4.82 |
|  | R | 1.64 | 1.64 | 6.02 | 2.41 |
| superior frontal | L | 3.28 | 3.28 | 2.41 | 3.61 |
|  | R | 6.56 | 3.28 | 4.82 | 3.61 |
| superior parietal | L | 6.56 | 1.64 | 3.61 | 1.20 |
|  | R | 1.64 | 0.00 | 2.41 | 1.20 |
| superior temporal | L | 1.64 | 3.28 | 1.20 | 3.61 |
|  | R | 3.28 | 1.64 | 2.41 | 4.82 |
| supramarginal gyrus | L | 4.92 | 1.64 | 3.61 | 2.41 |
|  | R | 3.28 | 3.28 | 2.41 | 3.61 |
| frontal pole | L | 1.64 | 1.64 | 4.82 | 1.20 |
|  | R | 4.92 | 1.64 | 2.41 | 2.41 |
| temporal pole | L | 4.92 | 1.64 | 2.41 | 1.20 |
|  | R | 4.92 | 3.28 | 2.41 | 2.41 |
| transverse temporal | L | 3.28 | 1.64 | 2.41 | 1.20 |
|  | R | 3.28 | 0.00 | 2.41 | 2.41 |
| insula | L | 1.64 | 0.00 | 2.41 | 1.20 |
|  | R | 4.92 | 1.64 | 1.20 | 4.82 |
| **Surface Area** | | | | | |
| banks superior temporal sulcus | L | 1.64 | 4.92 | 0.00 | 2.41 |
|  | R | 0.00 | 4.92 | 1.20 | 4.82 |
| caudal anterior cingulate | L | 0.00 | 3.28 | 0.00 | 6.02 |
|  | R | 0.00 | 6.56 | 0.00 | 3.61 |
| caudal middle frontal | L | 0.00 | 1.64 | 1.20 | 4.82 |
|  | R | 0.00 | 3.28 | 0.00 | 2.41 |
| cuneus | L | 1.64 | 3.28 | 3.61 | 1.20 |
|  | R | 0.00 | 4.92 | 3.61 | 1.20 |
| entorhinal cortex | L | 1.64 | 3.28 | 2.41 | 2.41 |
|  | R | 1.64 | 1.64 | 0.00 | 2.41 |
| fusiform gyrus | L | 3.28 | 4.92 | 2.41 | 2.41 |
|  | R | 0.00 | 4.92 | 1.20 | 2.41 |
| inferior parietal | L | 1.64 | 1.64 | 0.00 | 6.02 |
|  | R | 0.00 | 4.92 | 0.00 | 3.61 |
| inferior temporal | L | 1.64 | 3.28 | 0.00 | 3.61 |
|  | R | 3.28 | 4.92 | 1.20 | 2.41 |
| isthmus cingulate | L | 1.64 | 1.64 | 0.00 | 1.20 |
|  | R | 0.00 | 3.28 | 2.41 | 2.41 |
| lateral occipital | L | 4.92 | 4.92 | 3.61 | 2.41 |
|  | R | 0.00 | 3.28 | 1.20 | 1.20 |
| lateral orbitofrontal | L | 3.28 | 1.64 | 1.20 | 1.20 |
|  | R | 1.64 | 4.92 | 0.00 | 3.61 |
| lingual gyrus | L | 0.00 | 3.28 | 3.61 | 1.20 |
|  | R | 1.64 | 0.00 | 2.41 | 1.20 |
| medial orbitofrontal | L | 0.00 | 1.64 | 1.20 | 2.41 |
|  | R | 3.28 | 1.64 | 1.20 | 1.20 |
| middle temporal | L | 1.64 | 4.92 | 1.20 | 3.61 |
|  | R | 3.28 | 3.28 | 2.41 | 0.00 |
| parahippocampal | L | 1.64 | 3.28 | 1.20 | 4.82 |
|  | R | 0.00 | 1.64 | 2.41 | 2.41 |
| paracentral | L | 0.00 | 4.92 | 0.00 | 3.61 |
|  | R | 0.00 | 3.28 | 1.20 | 3.61 |
| pars opercularis | L | 0.00 | 3.28 | 2.41 | 3.61 |
|  | R | 1.64 | 1.64 | 2.41 | 2.41 |
| pars orbitalis | L | 1.64 | 4.92 | 1.20 | 1.20 |
|  | R | 0.00 | 1.64 | 1.20 | 2.41 |
| pars triangularis | L | 0.00 | 6.56 | 1.20 | 4.82 |
|  | R | 0.00 | 3.28 | 1.20 | 1.20 |
| pericalcarine | L | 0.00 | 4.92 | 1.20 | 2.41 |
|  | R | 1.64 | 3.28 | 2.41 | 1.20 |
| postcentral | L | 0.00 | 3.28 | 0.00 | 3.61 |
|  | R | 1.64 | 4.92 | 2.41 | 4.82 |
| posterior cingulate | L | 0.00 | 4.92 | 0.00 | 2.41 |
|  | R | 1.64 | 3.28 | 0.00 | 4.82 |
| precentral | L | 1.64 | 3.28 | 0.00 | 2.41 |
|  | R | 1.64 | 1.64 | 1.20 | 3.61 |
| precuneus | L | 1.64 | 1.64 | 1.20 | 3.61 |
|  | R | 1.64 | 4.92 | 2.41 | 7.23 |
| rostral anterior cingulate | L | 1.64 | 3.28 | 1.20 | 2.41 |
|  | R | 1.64 | 4.92 | 1.20 | 7.23 |
| rostral middle frontal | L | 1.64 | 4.92 | 2.41 | 2.41 |
|  | R | 3.28 | 0.00 | 3.61 | 1.20 |
| superior frontal | L | 1.64 | 1.64 | 1.20 | 3.61 |
|  | R | 0.00 | 3.28 | 2.41 | 3.61 |
| superior parietal | L | 0.00 | 3.28 | 0.00 | 3.61 |
|  | R | 0.00 | 8.20 | 3.61 | 2.41 |
| superior temporal | L | 0.00 | 1.64 | 0.00 | 4.82 |
|  | R | 0.00 | 3.28 | 2.41 | 1.20 |
| supramarginal gyrus | L | 0.00 | 4.92 | 0.00 | 6.02 |
|  | R | 3.28 | 3.28 | 2.41 | 3.61 |
| frontal pole | L | 0.00 | 4.92 | 3.61 | 2.41 |
|  | R | 0.00 | 1.64 | 2.41 | 2.41 |
| temporal pole | L | 1.64 | 1.64 | 2.41 | 0.00 |
|  | R | 1.64 | 3.28 | 1.20 | 3.61 |
| transverse temporal | L | 0.00 | 8.20 | 0.00 | 6.02 |
|  | R | 0.00 | 4.92 | 1.20 | 1.20 |
| insula | L | 1.64 | 1.64 | 2.41 | 2.41 |
|  | R | 1.64 | 3.28 | 2.41 | 1.20 |
| ^a^ significant two-proportion z-tests difference in infranormal z-scores between FEM and HI at P_FDR_ < 0.05; ^b^ significant two-proportion z-tests difference in supranormal z-scores between FEM and HI at P_FDR_ < 0.05; FEM = first-episode mania; Hemi = hemisphere; HI = healthy individuals; L = left; R = right. | | | | | |

**S4. Normative modeling**

We used the CentileBrain framework (<https://centilebrain.org/>) to generate sex-specific normative models for each of the FreeSurfer-extracted regional measures of cortical thickness (CT), Surface Area (SA), and Subcortical Volume (SV) (Table S1) estimated using Multivariate Fractional Polynomial Regression (MFPR) in a pooled sample of 37,407 healthy individuals (53.3% female) with an age range of 3-90 years [Ge et al., 2023]. A normative model for each morphometric measure was estimated using the following procedures: (i) data preparation: Sex-specific subsamples of each pooled sample were randomly split into a training subset (80%) and a test subset (20%) stratified by scanning site. In each subset, data were mean-centered after extreme values in each site-dataset were identified and removed using the interquartile range (IQR) method; (ii) site harmonization was implemented using ComBat-GAM [Fortin et al., 2017]; (iii) normative model of each neuroimaging measure was generated using MFPR implemented using the “mfp” package in R and the closed test procedure (known as RA2) to select the most appropriate fractional polynomial. Initially, models were trained using 5-fold cross-validation (5F-CV) in the corresponding sex-specific training subset with age being the only explanatory variable and then model parameters were tested in the corresponding sex-specific test subset. Model performance was evaluated using the Mean Absolute Error (MAE) and Root Mean Square Error (RMSE); the MAE is the average of the absolute differences (i.e., errors) between the predicted and the actual value of the outcome variable and the RMSE is the standard deviation of the prediction errors; (iv) model optimization involves the inclusion in the models of covariates pertaining to acquisition and global neuroimaging features (i.e., ICV for SV models, mean cortical thickness for CT models and mean cortical surface area for SA models. For each FreeSurfer-extracted measure, individualized z-scores were generated by subtracting the predicted value ($\hat{Y}$) from the observed value ($Y_{o}$) of that measure divided by the RMSE of the model.

**S5. G-brainAGE Computation**

Sex-specific models of global brainAGE (G-brainAGE) were selected amongst the best-performing models after systematically examining the impact of algorithm, site harmonization, age range, and sample size on brain-age prediction in a discovery sample of brain morphometric measures from 35,683 healthy individuals (age range: 5-90 years; 53.59% female) [Yu et al., 2024]. Based on our empirical data, the algorithm chosen was Support Vector Regression with Radial Function Kernal because of its resilience to outliers. The pre-trained models were tested for cross-dataset generalizability in an independent sample comprising 2,101 healthy individuals (age range: 8-80 years; 55.35% female) and for longitudinal consistency in a further sample comprising 377 healthy individuals (age range: 9-25 years; 49.87% female). The final models do not include site harmonization as the accuracy of age prediction from morphometry data was higher when no site harmonization was applied. The models were optimised when G-brainAGE was estimated after dividing our lifetime sample into two age-bins (5-40 years and 40-90 years), as this provided a better balance between model accuracy and explained age variance than any other alternative. These final model parameters were incorporated into CentileBrain [<https://centilebrain.org/#/brainAGE>], an open-science, web-based platform for individualized neuroimaging metrics. These models were also applied to the current study sample to compute G-brainAGE.

**S6. Local brainAGE computation**

We calculated L-brainAGE using the methods developed by Popescu and colleagues, 2021. Data from multiple cohorts were collated to create a discovery sample comprising 3463 T1-weighted MRI brain scans from healthy people (aged 18-90 years) while an independent sample of 692 healthy people was used as the hold-out test sample. Data from the discovery sample were randomly subsampled and split into a training (80%) and validation set (20%). The model parameters were determined in the training set and applied to the validation set. The training process involved entering the modulated GM and WM maps derived from SPM12 as input features to a convolutional neural network (CNN) that adopted a U-Net architecture (<https://github.com/SebastianPopescu/U-NET-for-LocalBrainAge-prediction>) [Ronnenberg et al., 2015]. The GM and WM images were split into overlapping 3-dimensional blocks of 523 voxels. The convolutional layers in the network used an isotropic 3×3×3 filter, convolved over the input image after which element-wise multiplication with the filter weights and subsequent summation was performed at each location. Subsequently, to allow for non-linear modeling, the obtained values were processed using an “activation function”; specifically, an activation function (i.e., LeakyReLu) with $\alpha=0.2$ was used. $LeakyReLu(\alpha)$ was defined as: $LeakyReLu(x) = max(x, 0) + min(x * \alpha, 0)$, which allows a small, non-zero gradient when the unit is not “active”. The convolution operation was also controlled by its stride, which is how many voxels are skipped after every element-wise weight multiplication and summation. The value of stride was set to 1. The algorithm used down-sampling which increases the effective field of view or “receptive field” of layers higher in the hierarchy. Down-sampling at each scale was implemented using two consecutive 3D 3×3×3 filter kernels with an initial number of channels set to 64, which was multiplied by 2 further down the down-sampling path. Down-sampling involved 2×2×2 average pooling. For the up-sampling part of the network, the down-sampling architecture was inverted, by replacing the down-sampling layers with 2×2×2 up-sampling layers. Each convolution used a squeeze-and-excite unit based on the Squeeze & Excite networks [Hu et al., 2018] to obtain age predictions over blocks of 123 voxels. Voxel-level mean absolute error (MAE) cost function on the output layer and two additional cost functions at the two other scales of the architecture were used: global average pooling followed by a dense layer to predict brain-age at block-level. The model was implemented in TensorFlow [Abadi et al., 2016].

Neuroimaging-based age-prediction is subject to regression dilution leading to a greater under- or over-estimate of age, the further away a sample is from the training set mean age. To account for this effect, Popescu et al used separate small batch (n=200) of participants randomly selected from the held-out dataset and obtained their voxel-level brain-age delta $A=\Delta_{i,v}$(i.e., predicted minus actual age), where $i$ indicates the $i$-th participant and $v$ the $v$-th voxel. Then participants were grouped according to their chronological aged into 5-year bins, with the first bin covering participants with a chronological age between 18-25 year. For each bin b the corresponding average voxel-level brain-age delta $\Delta_{b,v}$was calculated, which represents the average brain-age delta for that voxel given the chronological age interval. Subsequently, to de-bias the voxel-level brain-age delta for a new participant (e.g., from testing set), $\Delta_{j,v}$ the following formula was used:

${\tilde{\Delta}_{j,v}=\Delta}_{j,v}-\Delta_{b,v}$

The accuracy of the model parameters from the discovery dataset was then tested in the hold-out sample (n=692).

**S7. Group Differences in L-brainAGE**

With respect to L-brainAGE, case-control comparisons with age and sex as covariates were not significant at the designated P_FDR_<0.05. However, at a less conservative threshold (P<0.05, uncorrected, cluster size > 300 voxels), patients with first-episode mania showed reduced L-brainAGE in a cluster located within the right superior temporal gyrus (peak MNI coordinates: x=25.5, y=19.5, z=-40.5) (Supplementary Figure S1).


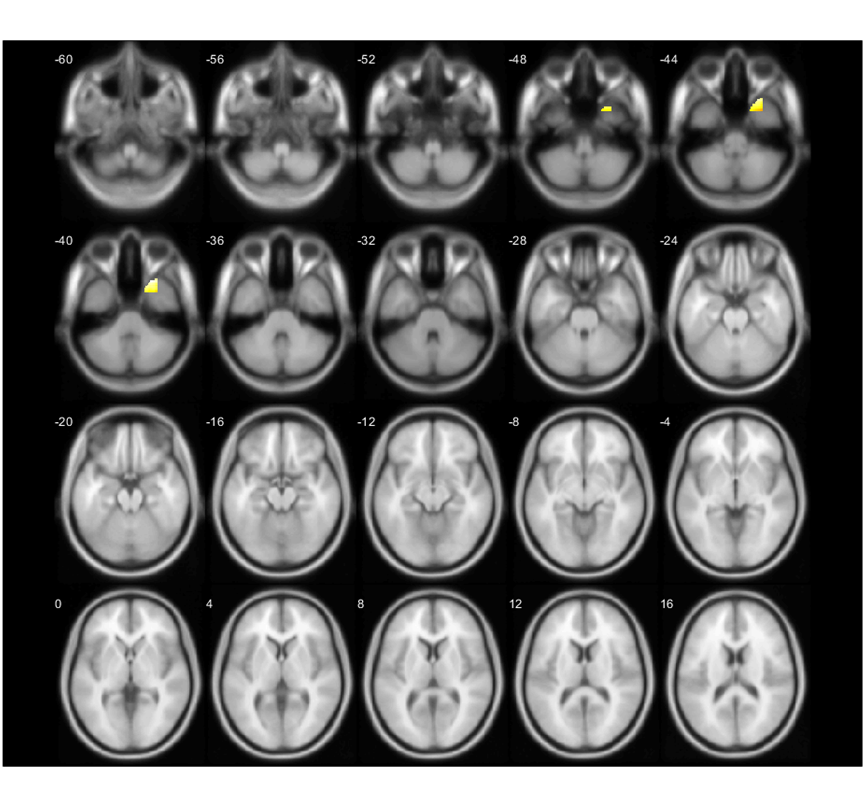


**Figure S1. Differences in L-brainAGE between healthy individuals and patients with first-episode mania.**

The figure depicts the T-value overlay of a statistically significant decrease in L-brainAGE (at P<0.05 uncorrected, cluster size>300 voxels) in patients as compared to controls. Images are displayed in neurological orientation.

**S8. References**

Abadi M, Barham P, Chen J, Chen Z, Davis A, Dean J, et al., editors. Tensorflow: A System for Large-Scale Machine Learning. 12th USENIX symposium on operating systems design and implementation (OSDI 16); 2016.

Ashburner J. A Fast Diffeomorphic Image Registration Algorithm. Neuroimage. 2007; 38(1):95-113.doi: 10.1016/j.neuroimage.2007.07.007.

Desikan RS, Ségonne F, Fischl B, Quinn BT, Dickerson BC, Blacker D, Buckner RL, Dale AM, Maguire RP, Hyman BT, Albert MS, Killiany RJ. An automated labelling system for subdividing the human cerebral cortex on MRI scans into gyral-based regions of interest. Neuroimage. 2006;31(3):968-80. doi: 10.1016/j.neuroimage.2006.01.021.

Fischl B, Salat DH, Busa E, Albert M, Dieterich M, Haselgrove C, van der Kouwe A, Killiany R, Kennedy D, Klaveness S, Montillo A, Makris N, Rosen B, Dale AM. Whole brain segmentation: automated labelling of neuroanatomical structures in the human brain. Neuron. 2002;33(3):341-55. doi: 10.1016/s0896-6273(02)00569-x.

Fortin JP, Cullen N, Sheline YI, et al. Harmonization of cortical thickness measurements across scanners and sites. *Neuroimage*. 2018;167:104-120. doi:10.1016/j.neuroimage.2017.11.024

Ge R, Yu Y, Qi YX, et al. Normative Modeling of Brain Morphometry Across the Lifespan using CentileBrain: Algorithm Benchmarking and Model Optimization. bioRxiv; 2023. doi: 10.1101/2023.01.30.523509.

Hu J, Shen L, Sun G, editors. Squeeze-and-Excitation Networks. Proceedings of the IEEE conference on computer vision and pattern recognition; 2018. doi:10.1109/TPAMI.2019.2913372

Le TT, Kuplicki RT, McKinney BA, Yeh HW, Thompson WK, Paulus MP; Tulsa 1000 Investigators. A Nonlinear Simulation Framework Supports Adjusting for Age When Analyzing BrainAGE. Front Aging Neurosci. 2018;10:317.

Popescu SG, Glocker B, Sharp DJ, Cole JH. Local Brain-Age: A U-Net Model. Front Aging Neurosci (2021) 13:761954. Epub 2021/12/31. doi: 10.3389/fnagi.2021.761954.

Ronneberger O, Fischer P, Brox T, editors. U-Net: Convolutional Networks for Biomedical Image Segmentation. International Conference on Medical image computing and computer-assisted intervention; 2015: Springer.

Yatham LN, Kauer-Sant'Anna M, Bond DJ, Lam RW, Torres I. Course and outcome after the first manic episode in patients with bipolar disorder: prospective 12-month data from the Systematic Treatment Optimization Program For Early Mania project. Can J Psychiatry. 2009;54(2):105-12. doi: 10.1177/070674370905400208.

Yu Y, Cui HQ, Haas SS, et al. Brain-age prediction: Systematic evaluation of site effects, and sample age range and size. *Hum Brain Mapp*. 2024;45(10):e26768. doi:10.1002/hbm.26768
